# Replacing statistical significance and non-significance with better approaches to sampling uncertainty

**DOI:** 10.3389/fphys.2022.962132

**Published:** 2022-09-05

**Authors:** Will G. Hopkins

**Affiliations:** Institute for Health and Sport, Victoria University, Melbourne, VIC, Australia

**Keywords:** Bayesian inference, confidence interval, effect magnitude, magnitude-based inference, sampling uncertainty, significance test

## Abstract

A sample provides only an approximate estimate of the magnitude of an effect, owing to sampling uncertainty. The following methods address the issue of sampling uncertainty when researchers make a claim about effect magnitude: informal assessment of the range of magnitudes represented by the confidence interval; testing of hypotheses of substantial (meaningful) and non-substantial magnitudes; assessment of the probabilities of substantial and trivial (inconsequential) magnitudes with Bayesian methods based on non-informative or informative priors; and testing of the nil or zero hypothesis. Assessment of the confidence interval, testing of substantial and non-substantial hypotheses, and assessment of Bayesian probabilities with a non-informative prior are subject to differing interpretations but are all effectively equivalent and can reasonably define and provide necessary and sufficient evidence for substantial and trivial effects. Informative priors in Bayesian assessments are problematic, because they are hard to quantify and can bias the outcome. Rejection of the nil hypothesis (presented as statistical significance), and failure to reject the nil hypothesis (presented as statistical non-significance), provide neither necessary nor sufficient evidence for substantial and trivial effects. To properly account for sampling uncertainty in effect magnitudes, researchers should therefore replace rather than supplement the nil-hypothesis test with one or more of the other three equivalent methods. Surprisal values, second-generation *p* values, and the hypothesis comparisons of evidential statistics are three other recent approaches to sampling uncertainty that are not recommended. Important issues beyond sampling uncertainty include representativeness of sampling, accuracy of the statistical model, individual differences, individual responses, and rewards of benefit and costs of harm of clinically or practically important interventions and side effects.

## Introduction

Read the abstract of any sample-based study and you will see that authors almost invariably use the data in their sample to make a claim about whether or not there is an effect. This dichotomization of outcomes appears to be a consequence of the widespread and often mandated use of statistical significance and non-significance, with which authors interpret *significant* as real, meaningful, worthwhile, important, useful, beneficial, harmful, or otherwise substantial, whereas they interpret *non-significant* as meaningless, worthless, useless, unimportant, inconsequential, or otherwise trivial. Non-significant is even sometimes presented as no effect whatsoever, the nil or zero in the null-hypothesis significance test (NHST), in which the null hypothesis is that there is no effect. (I will therefore refer to NHST as the *nil*-hypothesis significance test, to distinguish it from tests of other magnitudes.) Whether they understand it or not, authors are using statistical significance and non-significance as a method to account for uncertainty arising from sampling variation: another sample would give a different value of the effect (and a different *p* value for NHST), and it is only when samples are very large that the sample values would always be practically the same and therefore accurately represent the population or true value, provided of course that the sample properly represents the population.

Are authors justified in claiming that significant means the effect is real and non-significant means no effect? Such claims are consistent with the plain-English meanings of *significant* and *non-significant*. The design of NHST also leads authors to make such claims, because it is based on using a sample size that would give a reasonably high chance (usually 80%, the power of the study) of obtaining statistical significance (usually *p* < 0.05), when the true effect is the smallest important (sometimes referred to as the *minimal clinically important difference*). Unfortunately, statistical significance and non-significance do not directly address the evidence that an effect is substantial or trivial. Other approaches do, and as I will explain, they show that significance and non-significance are not fit for purpose.

The fundamental and irreparable problem with statistical significance and non-significance is the nil-hypothesis test: if you are interested in whether an effect is substantial or trivial, testing whether the effect could be nil or zero self-evidently misses the point. Instead, if you believe that hypothesis testing is the basis of the scientific method, you should test the hypotheses that the effect is substantial and trivial, then make decisions about magnitude based on rejection of the appropriate hypotheses. Alternatively, if you dislike the dichotomization of hypothesis testing and believe instead that estimation is the basis of empirical science, you should estimate the probabilities that the effect has substantial and trivial magnitudes, then make decisions based on threshold probabilities. You can even avoid making overt decisions and simply present qualitative and quantitative statistics representing either the range of possible effect magnitudes or level of evidence for or against effect magnitudes. In this article I will show that these alternative approaches are effectively equivalent, when they are understood in terms of the confidence interval or the sampling distribution from which it is derived. In the Discussion section, I also critique three more recent proposals for dealing with sampling uncertainty: surprisal values, second-generation *p* values, and the hypothesis comparisons of evidential statistics. The article is an updated version of a discussion paper on sampling uncertainty that I circulated to *Frontiers* and other journal editors in the disciplines of exercise and sport science (Hopkins, 2021a).

## The confidence or compatibility interval

**FIGURE 1:**
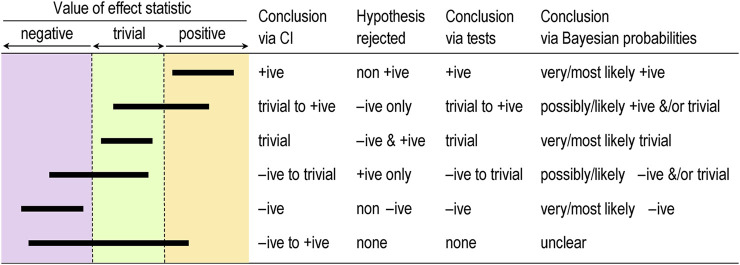
Conclusions about effects determined by coverage of the confidence or compatibility interval (CI), by tests based on rejection of one-sided interval hypotheses, and by Bayesian probabilities, for six qualitatively different dispositions of 90% CI (bars) relative to substantial and trivial magnitudes. +ive, substantial positive; −ive, substantial negative.

The best measure of sampling uncertainty is probably the confidence interval. The interval is usually interpreted in terms of precision of estimation, with larger samples producing narrower intervals that represent more precise estimates. This interpretation is the basis of a qualitative approach to sampling uncertainty promoted by Ken Rothman in his epidemiological texts (e.g., Rothman, 2012) and by psychologist Geoff Cumming in his “new statistics” (e.g., Cumming, 2014). These authors interpret the interval as a range of values of the effect, but they avoid describing the range as possible true values of the effect–an interpretation that requires a Bayesian analysis, as described below. The authors also offer little guidance on what level of confidence is appropriate, but Rothman’s examples feature 90% intervals three times more frequently than 95% intervals. Figure 1 shows the six different conclusions about the magnitude of an effect, depending on the disposition of the confidence interval in relation substantial and trivial magnitudes defined by the smallest important values. The resulting conclusion are properly phrased in terms of compatibility or incompatibility of those values with the data and model; for this reason, *compatibility interval* is perhaps a better term than *confidence interval* (Rafi and Greenland, 2020).

Rothman and Cumming emphasize that their method is a replacement for statistical significance. For example, Rothman (2012) states “Estimation using confidence intervals allows the investigator to quantify separately the strength of a relation and the precision of an estimate and to reach a more reasonable interpretation… In most instances, there is no need for any tests of statistical significance to be calculated, reported, or relied on, and we are much better off without them.”

## Tests of substantial and non-substantial hypotheses

The idea of testing whether an effect is substantial or trivial rather than nil has been promoted for many years in the guise of (non-)inferiority, (non-)superiority, equivalence, and one-sided interval-hypothesis testing (e.g., Allen and Seaman, 2007), but the approach is still rarely used. The compatibility interpretation of the confidence interval provides a straightforward way to understand how the tests work (Figure 1). If the interval falls entirely in, say, substantial positive values, non-positive values are not compatible with the data and statistical model, so the hypothesis that the effect is non-positive can be rejected. Conclusion: the effect is substantial positive (or strictly, not non-positive). With a 90% compatibility interval, the *p* value for the test (p_N+_) would be < 0.05, the exact *p* value being provided by the sampling distribution from which the compatibility interval is derived. If the interval falls entirely in trivial values, two one-sided hypotheses are rejected: the hypothesis that the effect is substantial positive and the hypothesis that the effect is substantial negative. Conclusion: the effect is trivial (or strictly, not substantial positive and negative). With a 90% compatibility interval, the *p* values for each test (p_+_ and p_−_) are both < 0.05, and the exact *p* values are provided by the sampling distribution. Compatibility intervals that include trivial magnitudes and substantial magnitudes of one sign imply rejection of the hypothesis of magnitudes of the other sign, and compatibility intervals that include substantial magnitudes of both signs imply rejection of no hypotheses. The correspondence between qualitative interpretations of compatibility intervals and the outcome of tests of substantial and non-substantial hypotheses should now be obvious.

Rejecting an hypothesis about a magnitude is decisive about the magnitude in a *necessary* sense. If the true effect is substantial, you must be able to reject the hypothesis that the effect is non-substantial at whatever chosen alpha level (*p*-value threshold) of the hypothesis test, although you might need a large sample size. Similarly, if the true effect is trivial, you must be able to reject the two substantial hypotheses. Rejecting hypotheses about magnitude is also decisive in a *sufficient* sense. Rejecting a substantial positive or negative hypothesis is sufficient to decide that the magnitude is not substantially positive or negative, with an error rate defined by the alpha of the test. Similarly, rejecting a non-substantial-positive or non-substantial-negative hypothesis is sufficient to decide that the true effect is substantial positive or substantial negative, and rejecting both substantial hypotheses is sufficient to decide that the true effect is trivial, with error rates defined by the alphas. Hypothesis testing is not guaranteed to be decisive for a given effect in a given study: rejecting the appropriate hypothesis (and thereby reaching the right conclusion) when the true effect is substantial or trivial will require a sample size that gives a compatibility interval narrow enough to exclude the hypothesized magnitude most of the time. I have addressed the issues of sample-size estimation with these tests elsewhere (Hopkins, 2020).

A problem with hypothesis testing, as with the compatibility interval, is choosing the appropriate alpha or compatibility level for a given kind of effect in a given setting. The Bayesian approach with probabilities of substantial and trivial magnitudes offers a solution to this problem.

## Probabilities of substantial and trivial magnitudes

For some researchers, dichotomization is an undesirable aspect of hypothesis testing: you conclude that the effect is definitely not something. Admittedly, the dichotomizing is softened somewhat by the up-front error rate represented by the *p*-value threshold. However, the *p* value represents evidence *against* a magnitude, and the test therefore does not lend itself easily to a more accessible expression of level of evidence *for* a magnitude. Bayesian analysis provides such evidence in the form of probabilities that the effect is substantial in a positive sense, substantial in a negative sense, and trivial (substantial in neither sense).

In a Bayesian analysis, the uncertainty in the true effect is defined by a posterior probability distribution of the true effect, which is derived by combining the sample data with prior belief or information about the uncertainty in the effect. A full Bayesian implementation is challenging, since a prior probability distribution has to be found and justified for every parameter in the statistical model used to derive the effect. All these parameter priors can be imagined coalescing into a single prior uncertainty in the true effect, which is then combined with the data. Greenland’s (2006) simplified Bayesian method uses this approach, which is at once more intuitive than a full Bayesian analysis and easily implemented (e.g., with a spreadsheet: Hopkins, 2019). The probabilities of substantial and trivial magnitudes are derived as the areas of the posterior distribution falling in substantial and trivial values.

Informative priors based on belief are difficult to justify and quantify, and the more informative they are, the more they are likely to bias the effect. They therefore offer the researcher an opportunity to bias the effect towards a *desired or expected* magnitude, by using a prior centered on that magnitude. Researchers can avoid these problems by opting for a prior sufficiently diffuse (weakly informative) that the posterior is practically identical to the original sampling distribution, which can then be interpreted directly as the probability distribution of the true effect. This approach to sampling uncertainty has been promoted by various authors (Burton, 1994; Shakespeare et al., 2001; Albers et al., 2018), including the progenitors of magnitude-based inference (MBI: Batterham and Hopkins, 2006; Hopkins and Batterham, 2016), also known as magnitude-based decisions (MBD; Hopkins, 2020). Formally, the approach is Bayesian assessment with a weakly informative prior taken to the limit of non-informative.

Some authors have claimed that MBI is not Bayesian, because a non-informative prior is not “proper” (it cannot be included in Bayesian computations, because it has zero likelihood for all values of the effect), and because such a prior implies a belief that unrealistically large values of the effect have the same likelihood (albeit zero) as realistic small values (Barker and Schofield, 2008; Welsh and Knight, 2015; Sainani et al., 2019). These criticisms have been addressed (Hopkins and Batterham, 2008; Batterham and Hopkins, 2015; Hopkins and Batterham, 2016; Batterham and Hopkins, 2019), most recently with the above argument that a realistic weakly informative normally distributed prior makes no practical difference to the posterior probabilities of the true effect for any reasonable sample size (Hopkins, 2019). For those with any lingering doubt, I would point out that even a full Bayesian analysis produces a posterior that is practically identical to the original sampling distribution, when the sample size is large enough to overwhelm the information in the prior. The sampling distribution can then be interpreted as the probability distribution of the true effect, yet the analysis and interpretation are still Bayesian. Equally, instead of making the sample size large, the prior can be made so weakly informative that it is overwhelmed by the data. The sampling distribution can then be interpreted as the probability distribution of the true effect, yet the analysis and interpretation are still Bayesian. There is no requirement with this argument for the weakly informative prior to be realistic, but as I have already stated, even realistic weakly informative priors make no practical difference with the small sample sizes typically encountered in exercise and sport science. It is therefore illogical for detractors to continue to state that MBI is not Bayesian.

With really small sample sizes, a weakly informative prior “shrinks” the posterior compatibility interval substantially and shifts it towards the middle of the prior. Researchers are welcome to apply such a prior, although in my view it is better to present the effect unbiased, with all its wide uncertainty, as a reminder to the researcher and the reader that the sample size in the study was woefully inadequate. Researchers may also wish to use reasonably informative priors to shrink the posterior even with the usual sample sizes. The resulting bias towards the prior will make the compatibility limits look more realistic, if that is a problem, but I would caution that the cost is downward bias and a resulting reduction of the chances of discovering a substantial effect.

It has also been claimed that MBI has a high Type-I or false-positive error rate (Welsh and Knight, 2015; Sainani, 2018). Those who make this claim interpret a possibly or likely substantial effect as a decisively substantial effect, then show that a high proportion of such effects are not statistically significant when sample sizes are small. There are two flaws with this claim. First, it is only when an effect is very likely substantial that the effect is considered decisively substantial (the compatibility interval falls entirely in substantial values). Secondly, as argued in this article, statistical significance is not a criterion for substantial. When errors are defined in terms of declaring a true trivial effect to be substantial and declaring a true substantial effect to be trivial, the error rates of the various forms of MBI were shown by simulation to be acceptable and generally superior to those of NHST for various effect magnitudes and sample sizes in controlled trials (Hopkins and Batterham, 2016). Interestingly, the same simulations showed that publication rates (of “clear” effects in MBI and statistically significant effects in NHST) and resulting publication bias of the various forms of MBI were also superior to those of NHST. All these findings applied not only to the usual sample size required for 5% significance and 80% power with NHST, but also to much smaller sample sizes: 10 + 10 in a controlled trial, when 50 + 50 were required for adequate precision with MBI and ∼150 + 150 were required for NHST. Naturally, small sample sizes often produce unclear outcomes, but these are not false positives or false negatives; rather, the conclusion is that more data are needed to resolve the uncertainty about the magnitude.

The most recent criticism of MBI is that it is misused by authors interpreting *possibly* and *likely* substantial as *decisively* substantial (Lohse et al., 2020). Close examination of the publications showed that most authors were not misusing MBI in this manner (Aisbett, 2020). Such misuse, when it occurs, should be easy to identify and correct during the process of peer review.

Magnitude-based inference goes further than its Bayesian predecessors by providing qualitative interpretations of the probabilities of substantial and trivial magnitudes and by suggesting different decision thresholds for the probabilities in non-clinical and clinical or practical settings (which can also be done in a full Bayesian analysis). Briefly, magnitudes in a non-clinical setting are considered decisive when they are very likely (probability > 0.95 or chances > 95%), corresponding to rejection of one or other non-substantial hypotheses (p_N+_ < 0.05 or p_N−_ < 0.05) or to rejection of both substantial hypotheses (such that p_+_ + p_−_ < 0.05); equivalently, the 90% compatibility interval falls entirely in substantial values or the 95% compatibility interval falls entirely in trivial values. See Figure 1. I established the mathematical equivalence of MBI and hypothesis testing by considering areas under normal probability distributions and error rates (Hopkins, 2020).

In a clinical or practical setting, an effect that is possibly beneficial (probability > 0.25 or > 25%) is considered potentially implementable, provided harm is most unlikely (probability < 0.005 or < 0.5%). Equivalently, the 50% compatibility interval overlaps beneficial values, or the beneficial hypothesis is not rejected (p_B_ > 0.25), while the 99% compatibility interval overlaps no harmful values, or the harmful hypothesis is rejected (p_H_ < 0.005). The different probability thresholds or compatibility intervals for benefit and harm in clinical MBI accord more importance to avoiding harm than to missing out on benefit. A less conservative version of clinical MBI, in which an effect is considered potentially implementable when the chance of benefit far outweighs the risk of harm (odds ratio > 66), does not have equivalent hypothesis tests.

If the probabilities of substantial and trivial magnitudes are such that no hypotheses are rejected, the outcome in MBI is described as unclear or indecisive, meaning that precision is inadequate and a larger sample size, better design, and/or better analysis are required. Once a substantial hypothesis is rejected, the qualitative probabilities of the other substantial and/or of trivial magnitudes are reported, as shown in Figure 1. The scale for these qualitative probabilities (25%–75%, possibly; 75%–95%, likely; 95%–99.5%, very likely; > 99.5%, most likely; Hopkins et al., 2009) is similar to but a little more conservative than that of the Intergovernmental Panel on Climate Change (Mastrandrea et al., 2010), who use their scale to communicate plain-language uncertainty in climate predictions to the public.

I have presented Bayesian analysis as an alternative to hypothesis testing, but as a reviewer pointed out, the Bayesian posterior credibility interval obtained with an informative prior can be used to test hypotheses (e.g., Gelman and Shalizi, 2013). In other words, the compatibility intervals shown in Figure 1 work equally well for testing hypotheses or for qualitative interpretation of magnitude when they are Bayesian posteriors. Researchers can therefore combine informative priors with data in a Bayesian analysis while maintaining the Popperian philosophy of falsification. The only problem is trustworthy quantification of informative priors.

## The nil-hypothesis significance test

An effect is statistically significant at the 5% level when the nil or zero hypothesis is rejected (*p* < 0.05); equivalently, the nil value of the effect is not compatible with the data and statistical model, so a 95% compatibility interval does not include the nil. The effect is statistically non-significant when the nil hypothesis is not rejected, so the 95% compatibility interval includes the nil.

**FIGURE 2:**
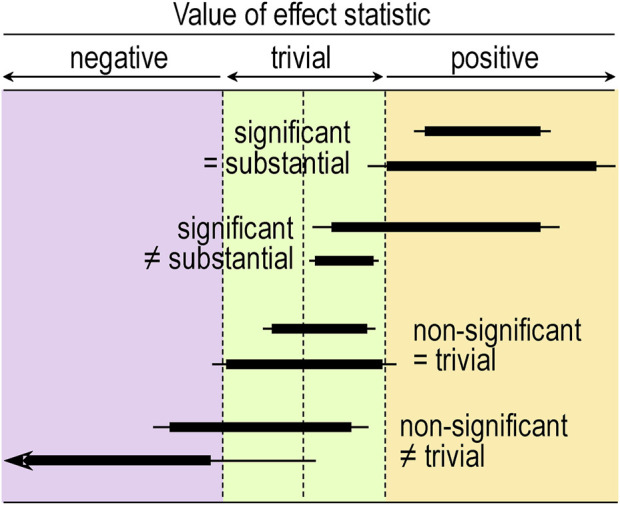
Compatibility intervals (thin bars, 95%; thick bars, 90%) illustrating significant effects where it would be appropriate (=) and inappropriate (≠) to conclude the effect is substantial, and non-significant effects where it would be appropriate and inappropriate to conclude the effect is trivial. The arrowhead indicates both compatibility intervals extending much further to the left.

If you allow that coverage of 90% compatibility intervals or the corresponding interval-hypothesis tests provide conclusive evidence about magnitudes, Figure 2 shows the scenarios where conclusions of substantial for significance and trivial for non-significance are appropriate: the 90% interval has to fall entirely in substantial or trivial values, respectively. Figure 2 also shows scenarios where these conclusions are not appropriate. These scenarios should convince you that significance is not sufficient for the effect to be decisively substantial (some significant effects could be trivial or even decisively trivial), while non-significance is not sufficient for the effect to be decisively trivial (some non-significant effects could be substantial or even decisively substantial). Two of the examples show that significance and non-significance are not even necessary respectively for decisively substantial and decisively trivial: decisively substantial can be not significant (the last example in the figure), and decisively trivial can be not non-significant, i.e., significant (the fourth example). In short, significant and non-significant are sometimes not the same as decisively substantial and decisively trivial.

The prevalence of appropriate conclusions about magnitude based on significance and non-significance in a given discipline will depend on the magnitude and uncertainty of effects relative to smallest important values. In a sample of studies related to athletic injury or performance at a recent sport-science conference, substantial was an appropriate conclusion for only 52% of significant effects, while trivial was appropriate for none of the non-significant effects, on the basis of coverage of 90% intervals or non-clinical MBI (Hopkins, 2021b). The proportion of significant effects that were potentially beneficial or harmful on the basis of clinical MBI was higher (65%), especially with odds-ratio MBI (87%), but again, none of the non-significant effects were decisively trivial with clinical MBI. I know of no other study where the misuse of significance and non-significance has been quantified in this fashion, but from my own experience, the misuse of significance and non-significance in peer-reviewed journals is no better than at this conference. As such, NHST should be “retired” (Amrhein et al., 2019).

## Discussion

A figure showing the disposition of a 90% compatibility interval relative to smallest important and other substantial magnitudes is the simplest tool for authors and readers to avoid making unrealistic conclusions about the magnitude of an effect and its uncertainty. The conclusion can be presented as the range in magnitudes represented by the lower and upper compatibility limits, and substantial can be further modified as small, moderate, etc. For example, an interval that begins in trivial values and ends in large positive values could be presented as trivial to large +ive (or trivial to large ↑, for factor effects), and the interval obligates the conclusion that the effect could be trivial to large positive and could not be substantially negative. The meanings of *could be* and *could not be* are defined by the level of the compatibility interval and can be expressed either in terms of rejection or failure to reject hypotheses or in terms of probabilities of the magnitudes. Indeed, some readers may prefer to see a more quantitative assessment of sampling uncertainty, so it seems reasonable for authors to also present hypothesis tests and probabilities of magnitudes. I have provided a relevant template for authors to include or cite in the methods section of their manuscripts, along with advice on the smallest and other important magnitude thresholds and on reporting effects in text, tables and figures (Hopkins, 2020). Use of probabilities is perhaps the best approach, because it represents a desirable move away from dichotomization and towards level of evidence, which could be described as *modest* or *some evidence* for a possible magnitude, *good evidence* for a likely magnitude, *very good evidence* for a very likely magnitude, and *strong evidence* for a most likely magnitude. Use of probabilities also allows better assessment of effects with clinical or practical relevance: a symmetric 90% interval does not capture the notion that strong evidence is needed against harm, while only modest evidence is needed for benefit. Furthermore, “you need only a modest probability of benefit, but you need a really low probability of harm” seems a more reasonable and accessible basis for proceeding to evaluate implementability than “you need failure to reject the hypothesis of benefit at some liberal *p*-value threshold and rejection of the harmful hypothesis at some conservative *p*-value threshold.”

I doubt whether the problems with NHST will be solved by editors allowing authors to provide the *p* value for NHST while prohibiting use of the terms *significant* and *non-significant*: most researchers will probably still think that *p* < 0.05 and *p* > 0.05 somehow provide additional or even criterion evidence for the presence and absence of effects. If *p* values are to be shown, they should be the Bayesian probabilities of substantial and trivial magnitudes, which as themselves or their complement (1 minus *p*) double as *p* values for hypothesis tests. The demise of NHST would also mean no more post-hoc tests conditioned on the statistical significance of predictors with more than two levels (e.g., group or time main effects or interactions). The magnitude and uncertainty of specific contrasts, pre-planned or otherwise, are what matter, regardless of the magnitude and uncertainty of any statistic summarizing the effect of all the levels (F ratios, variance explained, and so on). Any concern about an increase in error rate with multiple effects can be addressed by using higher levels of confidence for the compatibility intervals, smaller *p*-value thresholds for hypothesis tests, or more extreme probability thresholds for decisions.

In an effort to wean researchers off the dichotomization inherent in any hypothesis test, Greenland (2019) has promoted transformation of the *p* value of the test into an S or “surprisal” value, given by −log_2_(*p*), which is the number of consecutive head tosses of a fair coin that would have probability p. Researchers would present an S value rather than a *p* value, then assess a large S value as strong evidence against the hypothesized magnitude. I do not recommend S values for assessing sampling uncertainty, because they represent evidence *against hypotheses*, while the probabilities *for magnitudes* seem to me to be more accessible measures of evidence, especially when expressed as the plain-language terms *possibly, (un)likely, very (un)likely,* and *most (un)likely*.

A reviewer who rejected the manuscript on first review opined that “the second-generation *p*-value (SGPV) approach [is] more quantitative than the way offered by the author.” In one of the references cited by this reviewer to support this opinion (Stewart and Blume, 2019), there is the following succinct statement: “SGPVs measure the overlap between an uncertainty interval for the parameter of interest and an interval null hypothesis that represents the set of null and practically null hypotheses.” As such, SGPVs are similar to the *p* values of interval-hypothesis tests. Indeed, Lakens and Delacre (2020) have shown that the SGPV gives practically identical outcomes as equivalence testing achieved with two one-sided tests of substantial hypotheses “under optimal conditions,” but otherwise “the second generation *p*-value becomes difficult to interpret.” The *p* values for testing substantial and non-substantial hypotheses, and their complements (1 minus the *p* value) appear to be at least as good as SGPVs and probably retain the desirable qualities claimed for SGPVs (e.g., Stewart and Blume, 2019), when they are used to make inferences about whether the magnitude of an effect is substantial, non-substantial, or trivial. I therefore do not recommend the use of SGPVs.

I have limited this opinion piece to the issue of sampling uncertainty in settings where the form of the statistical model and the variables to include in it have been decided. In some research disciplines (e.g., ecology), models may be complex, their development is a primary consideration, and there are methods for choosing amongst competing models. Such methods are part of *evidential statistics*, an approach promoted as a successor to NHST and Bayesian statistics (e.g., Taper and Ponciano, 2016). Once a model has been selected with this approach, likelihood ratios are used to compare hypotheses about effects. In my view, formal comparison of hypotheses adds nothing to the necessary and sufficient evidence for magnitudes provided by the compatibility interval, the substantial and non-substantial hypotheses, and/or the relevant Bayesian probabilities.

I have also limited this opinion piece to a comparison of methods for making assertions about the likelihood of magnitudes of effects. In clinical or practical settings, where substantial magnitudes of effects of interventions represent benefit and harm, the likelihoods could be combined qualitatively or quantitatively with the perceived or actual rewards of benefit, the perceived or actual costs of harm, and with the likelihoods, rewards and costs of any beneficial and harmful side effects. These are considerations of decision theory that are beyond the intended scope of this article. My aim has been only to provide and justify better alternatives to NHST.

Whichever approach to sampling uncertainty you use, always be aware that your conclusions are usually about magnitudes of population mean effects. Effects on individuals are bound to be different, owing to individual differences and responses. You should include relevant subject characteristics as moderators in your statistical model or perform subgroup analyses to try to account for individual differences and responses, but there will always be residual errors representing unexplained variation, at least some of which arises from differences between individuals. The residual error can be used to make probabilistic assertions about the effects on individuals in sample-based studies (Hopkins, 2018; Ross et al., 2019) and when monitoring individuals (Hopkins, 2017).

Finally, a conclusion, decision or probabilistic statement about the magnitude of an effect derived from a sampling distribution is conditioned on assumptions about the data and the statistical model (Rafi and Greenland, 2020). The way in which violation of these assumptions could bias the outcome should be discussed and, where possible, investigated quantitatively (Lash et al., 2014). A straightforward method is sensitivity analyses, in which the width and disposition of the compatibility interval relative to smallest importants are determined for realistic worst-case violations (Hopkins, 2021b).
